# Preventing Emotional Dysregulation: Acceptability and Preliminary Effectiveness of a DBT Skills Training Program for Adolescents in the Spanish School System

**DOI:** 10.3390/ijerph19010494

**Published:** 2022-01-03

**Authors:** Xavier Gasol, María Vicenta Navarro-Haro, Isabel Fernández-Felipe, Azucena García-Palacios, Carlos Suso-Ribera, Miquel Gasol-Colomina

**Affiliations:** 1 Borderline Personality Disorder Institute Foundation, 08221 Terrassa, Spain; xerogatzol@gmail.com (X.G.); miquel_gasol@hotmail.com (M.G.-C.); 2 Department of Psychology and Sociology, University of Zaragoza, 44003 Teruel, Spain; 3 Instituto de Investigación Sanitaria de Aragón (IISA), 50009 Zaragoza, Spain; 4 Department of Basic Psychology, Clinical Psychology and Psychobiology, Universitat Jaume I, 12071 Castellón de la Plana, Spain; fernandi@uji.es (I.F.-F.); azucena@uji.es (A.G.-P.); susor@uji.es (C.S.-R.); 5 CIBER of Physiopathology of Obesity and Nutrition (CIBEROBN), ISCIII CB06/03/0052 Instituto Salud Carlos III, 28029 Madrid, Spain

**Keywords:** prevention, adolescents, secondary school, emotion regulation, transdiagnostic approach, evidence-based psychological interventions, dialectical behavior therapy

## Abstract

Emotional dysregulation is a key factor in the development and maintenance of multiple disabling mental disorders through a person’s lifespan. Therefore, there is an urgent need to prevent emotional dysregulation as early as possible. The main aim of this study was to evaluate the acceptability and preliminary effectiveness of an adapted Dialectical Behavior Therapy Skills Training program for Emotional Problem Solving in Adolescents (DBT STEPS-A) during secondary school. The sample included 93 adolescents (mean age = 12.78; SD = 0.54; and 53% female) studying in their 2nd year of secondary school in a public center in Catalonia (Spain). Measures of acceptability, difficulties of emotional regulation, mental health problems, and life satisfaction were completed before and after participation in the DBT STEPS-A program during one academic year. The majority of students rated the program as useful (64%) and enjoyed the classes (62%) and 48% of them reported practicing the newly learned skills. Statistically significant improvements were revealed in some emotional regulation-related variables, namely the number of peer problems (*p* = 0.003; d = 0.52) and prosocial behaviors (*p* < 0.001; d = −0.82). Although non-significant, the scores in the remaining outcomes indicated a general positive trend in emotional dysregulation, mental health, and life satisfaction. The adapted DBT STEPS-A was very well-accepted and helped overcome some emotional regulation difficulties in Spanish adolescents.

## 1. Introduction

Difficulties in emotional regulation play a key role in the development and maintenance of numerous emotional disorders [1]. These emotional regulation problems also have long-term consequences if untreated. A study revealed that adults who experienced emotional regulation problems (attention problems, anxiety, depression, and aggressive behavior) during adolescence were more likely to be diagnosed with anxiety disorders, mood disorders, and disruptive behavior disorders 14 years later [2]. In addition, emotional dysregulation has been positively associated with devastating consequential outcomes, such as diagnosis of borderline personality disorder (BPD) [3] and self-harm and suicidal behaviors [4], which have now become the third leading cause of death in 15- to 19-year-olds, according to the World Health Organization [5]. Not surprisingly, it has been suggested that mental health prevention plans should focus primarily on the psychological well-being and mental health of adolescents [6]. To prevent severe and persistent mental disorders in the future, it is urgent to develop programs that address emotional dysregulation as early as possible [3].

Schools are considered adequate environments for the implementation of mental health promotion programs, as they serve as a natural and accessible way to reach young people [7]. Students are under a lot of stress. Some are facing mental health issues that need intervention by mental health professionals, and all young individuals will eventually suffer stressful situations that may interfere with their functioning [8,9]. Therefore, offering support to the students in the school environment may help them cope and practice new skills with guidance, in an attempt to reduce emotional difficulties [7,10]. A review of school-based mental health interventions has highlighted the need for more research to identify mental health disorders in children as a priority to maximize the effectiveness of school-based interventions [11]. Including mental health services into the school system was reported as a key factor in this direction, whereby integrative strategies would increase access to mental health support when required [12]. Despite all these benefits, using schools as the center for mental health services may have some limitations, as there is typically limited access to trained mental health professionals [10].

Dialectical Behavior Therapy (DBT) [13,14] is an integrative evidence-based treatment that was originally developed for adults at risk of suicide and later proved to be an effective treatment for BPD, a disorder characterized by pervasive emotional dysregulation. Because DBT is a multi-component, comprehensive, and flexible treatment approach, this intervention has been recently applied to other populations of adults and adolescents with a wide range of emotional regulation difficulties. Therefore, DBT has been recognized to be a transdiagnostic treatment strategy to address emotional dysregulation [15]. In addition to the encouraging results of DBT for adolescents with suicidal behaviors [16,17], a review reported that DBT is a promising intervention to treat young people with different problems related to emotional dysregulation, such as comorbid depression, bipolar disorder, maladaptive eating behaviors, and impulsive and aggressive behaviors [18]. The review’s findings suggest that DBT can be implemented in different contexts, such as outpatient, inpatient, residential, and community settings. In fact, an application of DBT to prevent emotional regulation difficulties in adolescents has been developed and it is currently being evaluated (DBT Skills Training for Emotional Problem Solving for Adolescents; DBT STEPS-A) [19]. The program consists of a social–emotional learning curriculum developed to teach decision-making skills and coping strategies for particularly emotionally stressful moments. These skills can be used for both mild and severe emotionally activating situations that have been associated with emotional dysregulation, such as academic pressures; alcohol and drug use; peer, family and romantic relationships; suicidal and self-injurious behaviors; physical and sexual abuse; victimization; and the perpetration of bullying [10].

With respect to the effectiveness of DBT programs for adolescents in the educational system, Zapolski and Gregory [20] conducted a pilot study of a DBT skills training group for 53 high school students. The pre–post-results of the study indicated that the implementation of the program in schools was feasible and showed preliminary evidence of its effectiveness to decrease the likelihood of young individuals engaging in risky behaviors due to positive mood. On the other hand, regarding the specific adaptation of the DBT for adolescents during school (DBT STEPS-A) [19], to our knowledge only two studies with adolescents have been published. A pilot implementation of this program was conducted by Flynn et al. [21] at the beginning and end of one academic year with 72 adolescent girls (aged 15–16) from two schools in the south of Ireland. The participants received either a shorter (22-week) DBT STEPS-A treatment program or no intervention (control group). Emotional symptoms, dysfunctional coping, and use of DBT skills were analyzed. The findings indicated that for the participants in the DBT STEPS-A, their conditions showed a statistically significant reduction in emotional symptoms and internalizing problems, and effect sizes were large. In another recent study, a classroom guidance curriculum following DBT STEPS-A was evaluated in 94 ninth-grade students (42 treatment and 52 control) enrolled in a rural high school. Pre- to post-test changes indicated a treatment effect on social resilience and emotional regulation difficulties, as well as a good understanding and acceptance of DBT skills [22]. Finally, a recent quasi-experimental study conducted in Mexico evaluated the impact of the DBT STEPS-A on adult Spanish university students (n = 89) by comparing four groups (treatment as usual: TAU; TAU + DBT STEPS-A, DBT STEPS-A, and a group of no intervention). The results showed statistically significant differences in favor of the TAU + DBT STEPS-A group on depression, anxiety, and some difficulties in emotional regulation, namely in acceptance and goals, as well as in the severity of mental health problems [23]. 

Even though a few adaptations of DBT have shown encouraging findings in relation to emotional dysregulation in adolescents during school, rigorous studies are needed to replicate and enhance the reliability of the results. Furthermore, although one study has evaluated the effectiveness of the DBT STEPS-A program in a sample of Spanish-speaking young adults, to the best of our knowledge, there are no studies that have investigated the DBT STEPS-A specifically for adolescents in Spanish-speaking countries. The main goal of the study was to evaluate the acceptability and preliminary effectiveness of an adapted DBT STEPS-A program during one academic year in a sample of students attending secondary school in a public educational center in Catalonia (Spain). Our main hypothesis was that the adapted DBT STEPS-A treatment would be well accepted by students, as measured by an anonymous satisfaction survey combining quantitative and qualitative data at the end of the program. Secondly, after the implementation of a quasi-experimental design, we expected that the program would result in significant improvements in emotional dysregulation, psychopathology (difficulties in mental health features), and satisfaction with life.

## 2. Materials and Methods

### 2.1. Participants

The total sample was composed of 93 students from four school groups (range of 24–27 students per group) studying in their second year of secondary school in a public school in Terrassa (Barcelona, Spain). Given that the curriculum is designed at a universal level, all the students of the four groups received the program, which was implemented during the academic schedule. The characteristics of the sample are detailed in the results section. Because this is a pilot study with a convenience sample, no a priori sample size calculation was made.

### 2.2. Instruments

Acceptability and skills practice: A 12-item anonymous survey developed by the research team was administered to measure acceptability of the adapted DBT STEPS-A intervention. Questions regarding DBT STEPS-A skills practice were also included. In addition to the quantitative questions, four qualitative questions were added. The survey questions are detailed in the results section. This survey was evaluated at the end of the program.

Satisfaction with life: The Satisfaction with Life Scale (SWLS) [24], which has been validated into Spanish, was also included in the assessment protocol [25]. This is a short 5-item instrument that uses a 7-point Likert-style response scale that was designed to measure global cognitive judgments of satisfaction with one’s life. This scale has been used widely as a measure of the life satisfaction component of subjective well-being, as well as an indicator of quality of life. Scores between 5 and 9 are interpreted as showing extreme dissatisfaction with life, whereas scores between 31 and 35 indicate extreme satisfaction. The alpha coefficient for the scale has ranged from 0.79 to 0.89, indicating that the scale has high internal consistency [26]. Good psychometric values have been also found in a Spanish validation with adolescents [27]. Reliability results of the study sample were also good in the present study (Cronbach’s alpha of 0.75).

Mental health difficulties and strengths: The Strengths and Difficulties Questionnaire (SDQ) [28] was administered. The official Spanish translation of the SDQ was used [29]. The SDQ consists of 25 items measuring both mental health problems and competencies in childhood and adolescence. Items can be allocated to 5 subscales of 5 items each. These are emotional symptoms, conduct problems, hyperactivity/inattention, peer problems, and prosocial behaviors. Each item is scored on a 3-point scale where 0 = ‘not true’, 1 = ‘somewhat true’, and 2 = ‘certainly true’. A total difficulties score is generated from the sum of the four problems sub-scales. The items for prosocial behaviors (5 items) are not included in the total difficulties score. A higher total difficulty score indicates a greater likelihood of significant problems, whereas higher scores in the prosocial scale indicate more prosocial behaviors or strengths. The scale has demonstrated adequate psychometric properties across different cultural populations [30,31,32]. Reliability results of our study were: Cronbach’s alpha of 0.70 for the total difficulties score, 0.75 for the emotional problems scale, 0.68 for the conduct problems scale, 0.79 for the hyperactivity scale, 0.69 for the peer problems scale, and 0.70 for the prosocial behavior scale.

Difficulties in Emotional Regulation: The Difficulties in Emotional Regulation Scale (DERS) [33] was selected for this study. The version adapted and validated into Spanish by Hervás and Jódar [34] was used. The DERS is a scale designed to evaluate relevant aspects of the emotional regulation process in which difficulties may exist. When adapting the scale to Spanish, the authors reduced the items from 36 to 28 and considered only five subscales: emotional inattention, emotional confusion, emotional rejection, daily interference and emotional uncontrol, and a total score. The instrument is answered in a Likert format (1 = almost never, 5 = almost always), where a higher score indicates more difficulties in emotional regulation. For the Spanish version, the internal consistency of the subscales ranged from 0.78 to 0.91, and 0.93 was obtained for the total score [34]. Good psychometric findings were also found in a sample of Spanish adolescents (α = 0.71–0.88) [35]. Reliability results of the present study sample also showed good internal consistency (Cronbach’s alphas were: 0.89 for total score, 0.81 for emotional inattention, 0.80 for emotional rejection, 0.86 for daily interference, and 0.87 for emotional uncontrol), except for the emotional confusion subscale (lower than 0.60), which was not used in the current study.

### 2.3. Procedures

A public school from Terrassa (Barcelona) was selected to conduct this pilot study within a larger co-funded project (European Regional Development Fund, FEDER Program of Catalonia 2014–2020, and by the Diputació de Barcelona, for the project “E-Mocional.reg” in the context of territorial specialization and competitiveness projects (PECT) “Health Care Innovation Lab Orbital 40”), which aimed to evaluate efficacy of the DBT STEPS-A program in different schools of Terrassa, Barcelona (Spain). The ethical committee of the Hospital General of Catalonia (Sant Cugat, Barcelona, Spain) approved the study procedures (2017/05-PSQ-HGC).

The head teacher and the schoolteachers were informed about the study by the research team. The teachers received an introductory training course on DBT STEPS-A (8 h) by two intensively trained DBT therapists before conducting the study. The adapted DBT STEPS-A program in the public secondary school was conducted by two clinical psychologists working at the Personality Disorders Unit of the Hospital General of Catalonia (Sant Cugat del Vallès, Barcelona). One of the psychologists (not an author on this manuscript) was trained in the United States of America (USA) by the treatment developers of the DBT-STEPS-A, and the other clinical psychologist (X.G.) was trained by another psychologist trained in the USA (M.V.N.-H.). Trained teachers participated as observers and co-teachers during the program to learn how to teach the skills during the following academic year.

Adolescents and their caregivers were informed of the study in-person at the beginning of the academic year (September 2019) and, if they were interested in participating, caregivers signed an informed consent to accept the participation of the adolescent. The instruments detailed above were administered via an online survey before (September 2019) and after the program (November 2020). The academic course usually ends in June and students have a vacation period until September. However, due to the COVID-19 restrictions, the program was interrupted for three months starting from March 2020. Therefore, the academic and research team decided to extend the program for three months in the following academic year, so the students finished the DBT STEPS-A sessions by the end of November 2020, as opposed to June as originally planned. During the COVID-19 lockdown, the students were sent a set of video pills to help them review and practice the skills that they had learned until lockdown.

All the students received the adapted DBT STEPS-A lessons during the academic schedule. Previously, the head teacher and the teachers discussed the best strategy to include the DBT STEPS-A program into the course curriculum. Due to similarities in the general learning goals, the academic team decided to include this program in the context of the “Ethical Values” course.

### 2.4. Intervention

Adapted DBT STEPS-A [19]: The DBT STEPS-A is a social–emotional learning curriculum developed to teach adolescents (12–19 years old) decision-making skills and coping strategies, especially in emotionally stressful situations. The curriculum is designed to be applied at a universal level (i.e., to all the students independently of if they have mental disorders or not) to increase the students’ emotional resilience and decrease emotional dysregulation. The foundation of the curriculum is based on Dialectical Behavior Therapy (DBT) [13], which provides evidence-based strategies to help students learn and practice different emotional regulation skills. The curriculum is divided into an orientation section and four modules: mindfulness (repeated between modules), distress tolerance, emotional regulation, and interpersonal effectiveness. The program consists of 30 weekly lessons during one academic year. The lessons are manualized (accompanied by student worksheets) and structured in 50 min periods of class. The curriculum also includes 3 tests that evaluate specified knowledge and skills applied in different situations. A skills diary is also included. This is completed by the students weekly and collected by the teachers. The lessons are structured as follows: (1) Mindfulness exercise (5 min); (2) short period to review the assigned tasks (10 min). The revision of the tasks is done in pairs or in trios, so that the teenagers support and teach strategies to each other. (3) Teaching a new skill and the contexts in which they should be used (30 min). Each new skill is accompanied by examples adapted to be used by teenagers. (4) Finally, the lesson ends with homework assignments related to the new skill taught during that lesson (5 min). 

Although the length, contents, and structure of the program were respected, some adaptations were done from the original program. Firstly, the contents were translated into Spanish by two intensively trained DBT clinicians. Secondly, the lessons were taught by clinical psychologists instead of teachers. Teachers were observers and, if needed, acted as co-teachers. The lessons were taught by the psychologists to train teachers so that they could apply the program in the following year. Finally, some of the contents were shortened for time reasons. The adapted DBT STEPS-A 30-lesson curriculum is presented in Table 1. 

### 2.5. Statistical Analysis

With respect to the quantitative data, descriptive statistics were calculated for demographic characteristics, satisfaction with the program, and effectiveness outcomes. To compare statistical differences between pre- and post-intervention instrument scores, a non-parametric signed-rank Wilcoxon test was conducted because some of the data did not show homogeneity of variances and/or a normal distribution. A *p*-value of ≤0.01 was considered statistically significant. Effect sizes were calculated with Cohen’s d. 

On the other hand, to analyze the qualitative data, a replication process based on the principles of thematic analysis was used [36]. First, the researchers conducted repeated readings of the transcripts where extracts from these transcripts were marked and coded with keywords. Codes that belonged to the same topic and were related to each other were grouped for the identification of the topics addressed in the satisfaction survey and were analyzed. Two researchers conducted the analyses independently to improve the quality of the study. These two researchers then discussed the coding and interpretation of the codes until a consensus was reached. The other authors also performed the reading and analysis of the transcript.

## 3. Results

### 3.1. Participant’s Characteristics

The total sample was composed of 93 adolescents studying in their second year of secondary school. The participants’ age ranged from 12 to 15 years (their mean age was 12.78 years; SD = 0.54) and the gender distribution was 53% females and 47% males. Although 93 students completed the pre-assessment measures, only 51 of them completed the post-assessment as well. The reasons for non-completion varied: 15 students were at home the day of the post-assessment due to COVID-19 restrictions (e.g., quarantine), 14 survey responses were not valid due to incompleteness, and 13 students preferred not to answer. 

### 3.2. Acceptability of the Adapted DBT STEPS-A

#### 3.2.1. Quantitative Results

As presented in Table 2, 64% of the students rated the course as useful for their life and 62% of them enjoyed the classes. Most students reported good results for the teachers’ performance with ratings either remarkable (34%) or outstanding (48%). The majority of students rated the emotional regulation course as good (40%) or remarkable (40%). Regarding the use of skills, around half of the students (48%) used them from the beginning of the course. As detailed in Table 2, considering the ratings of self-evaluation by the students, attention and participation in class, as well as respect for peers, teachers, and content, was generally satisfactory.

#### 3.2.2. Qualitative Results

Questions regarding the qualitative variables of the satisfaction survey are detailed in Table 3. The qualitative results obtained from these questions were divided into three topics, as follows:

1. Pros and Cons: Different pros and cons about the emotional regulation training program were found in the sample of adolescents. On the one hand, the program was perceived positively due to the repertoire of skills learned. Being able to communicate and express thoughts and emotions with others, increasing pleasant emotions, the usefulness of these skills, and the participation in activities during the program were the most frequently reported benefits. What the participants liked most about this program was: *“that we can learn about ourselves and learn ways to regulate our emotions in everyday situations”,* as well as *“that we can express our feelings”*, and *“skills are interesting and help us to feel better”*. In addition, another participant commented that they liked *“when we do activities in which we can participate and we all laugh a lot”.* On the other hand, some of the negative comments regarding the program included the perceived amount of homework that the students were asked to do, the difficulties in performing the Mindfulness skills, and the theoretical aspects of the class. In terms of homework and theoretical classes, the participants reported: *“I didn’t like doing homework because it was difficult”* and *“sometimes there is a lot of theory and sometimes it takes a long time”.* On the other hand, regarding Mindfulness, the concerns were that *“maybe there are people who like it, but I don’t fit in that group”* and *“staring at something or being with my eyes closed makes me dizzy”*;
2. Areas for improvement: Doing practical exercises during the theoretical classes was an important aspect in the perception of program satisfaction. The participants reported that they would have liked more practical exercises in class, instead of written exercises, as well as a greater variety of examples to be included into the theoretical explanations. The teenagers’ comments were as follows: “*more doing things instead of writing things down”, “more participation and less theory, more everyday examples”, “do more activities and examples”, “more participation activities”*, and *“we should do more practices by standing in front of the class”*;
3. Participants’ general opinions: Some of the participants reported that the emotional regulation skills program helped them to make decisions and manage problems, to regulate anger and anxiety, to understand emotional reactions, to know the process of emotional regulation, and to resolve interpersonal conflicts. The participants mentioned that “*it has helped me to relax when I get angry”, “the truth is that there are times when the course has helped me a lot, as I am a guy who gets nervous quickly, and the breathing and getting into the cold water was very useful”*, and “*the course has helped me solve and manage problems”.* One of the participants said that *“this course has helped me a lot to understand reactions that I myself had and did not know why; with this course I have understood that feelings cannot be controlled, but they can be regulated”.*

Furthermore, several adolescents have referred to the use of skills during the COVID-19 restrictions. Specifically, they commented on the utility of the skills in regulating their emotions: *“This lockdown has been a bit hard. Although I am an indoor person, staying with my parents 24/7 for 3 months has made it much easier for us to get angry, and, actually, practicing emotional regulation has saved me a lot of trouble with them”* and “*during the lockdown, the course has helped me a lot to relax and to not stress too much”.*

### 3.3. Preliminary Effectiveness of the Adapted DBT STEPS-A

Table 4 shows the descriptive statistics (means and standard deviations) of the study variables before (pre-test) and after (post-test) the implementation of the program. Mean differences, significance levels, and effect sizes for all the study variables are also calculated. 

With respect to the adolescents’ Satisfaction with Life Scale (SWLS), as seen in Table 4, mean scores at pre- and post-test correspond with cut-offs 15–19 [24], which can be interpreted as “slightly dissatisfied” in both time points. There was a non-significant increase in the SWLS from pre- to post-intervention. 

Regarding mental health difficulties (SDQ), pre- and post- mean scores on emotional, conduct, hyperactivity/inattention problem subscales, as well as total difficulties score correspond with cut-offs “close to average” [24], thus indicating that clinically significant problems in this area were not likely to be significant. These scores decreased after the intervention, but not significantly (all *p* < 0.05) and effect sizes were small (Cohen’s d below 0.20). A significant effect, particularly a decrease, occurred in peer problems from pre- (cut-off 3 “slightly raised”) to post-test (cut-off 0–2 “close to average”) with a medium effect size (*p* = 0.003; d = 0.52). In the same line, a statistically significant increase from pre- (cut-off 6 “slightly lowered”) to post-intervention (cut-off 7–10 “close to average”) in prosocial behavior was found and this effect was large (*p* < 0.001; d = −0.82). 

Finally, in relation to the Difficulties in Emotional Regulation Scale (DERS), the means of the DERS total score in our sample were slightly above the normative means in Spanish samples [34]. The results show that there was a non-significant decrease in total difficulties of emotional regulation at post-intervention (Table 4). Mean scores for emotional inattention, emotional rejection, emotional uncontrol, and daily interference subscales were also slightly above the normative mean [34] and they improved, but not significantly, after the intervention. 

## 4. Discussion

The main aim of this research study was to evaluate the acceptability and preliminary effectiveness of an adapted DBT STEPS-A program, in order to prevent emotional dysregulation and increase resilience at a universal level in a Spanish sample of students attending secondary school in a public educational center. To our knowledge, there are only two published studies that have evaluated the effectiveness of a DBT STEPS-A program for adolescents in the school system. The main findings of these studies suggest that this program has been well accepted by students and that the program might help reduce emotional dysregulation and improve social resilience in adolescents at a universal level. In this regard, research shows that when interventions are delivered at a universal level, relatively more severe and at-risk young persons may be screened for mental health concerns and provided with support [7,37]. Therefore, the general goal of the project was to include all the students in the classes to reach a wider range of problems and people in need. In addition to this, we intended to evaluate, for the first time, the acceptability and effectiveness of a DBT STEPS-A program for Spanish adolescents in the school system.

We hypothesized that students would show good acceptance of the adapted DBT STEPS-A program, as measured by a satisfaction survey. The results of this study showed that most adolescents thought that the emotional regulation course was useful for life, and they enjoyed the classes. Students also rated teacher performance and the course as remarkable. In addition, half of them have used the learned DBT STEPS-A skills since the course started. These findings are interesting since only one study evaluating DBT skills-use found good feasibility outcomes [22]. Including a measure of DBT skills-use such as the DBT Ways of Coping Checklist (DBT-WCCL [38]) for futures studies would help provide evidence of the specific DBT skills used by the adolescents. Furthermore, additional acceptability results were provided by the comments of the students regarding the benefits and areas of improvement in the qualitative questions. In general, the students reported several benefits of the DBT STEPS-A program. The skills helped them to understand, express, and regulate their emotions in everyday life situations, as well as to solve and manage their problems. On the other hand, it is important to emphasize that some students used the skills during the COVID-19 pandemic lockdown and reported that the skills were useful to regulate their emotions in their natural environment. These are relevant findings, since they suggest that the reported benefits fit with the aim of the program (i.e., to teach decision-making skills and coping strategies for emotionally stressful situations), and also that the students practiced not only at school but also in their personal lives, thus suggesting that the DBT program successfully helps to generalize skills to the natural context [14]. Regarding the disadvantages, most students agreed that reducing the theoretical content and adding more practical exercises were aspects that needed to be improved. These qualitative results are important since they can inform program implementation and refinement. 

Secondly, we expected that the adapted DBT STEPS-A would lead to significant differences in emotional dysregulation dimensions (difficulties of emotional regulation), general symptoms of psychopathology (difficulties in mental health features), and satisfaction with life when comparing before and after the program. Regarding the difficulties in emotional regulation (as measured by the DERS), there was a pre- to post-test decrease in all the variables (total score, emotional inattention, emotional rejection, lack of emotional control, and daily interference subscales). However, no statistically significant differences were found in these DERS scales. These findings are in line with the results of the study by Flynn et al. which indicated that although difficulties in emotion regulation improved after a briefer DBT STEPS-A program, there were no statistically significant differences [21]. However, two adaptations that added other components to the DBT STEPS-A for adolescents [22] and young adults [23] did find statistically significant decreases in these difficulties. Concerning the changes in emotional, conduct, and hyperactivity/inattention mental health problems, as well as the total difficulty scores of the SDQ, the findings showed that pre- and post-scores were “close to average” compared to normative samples and that a non-significant small reduction on these outcomes was found after the program. The social variables of the SDQ showed a different pattern. Firstly, peer problem scores at pre-test were “slightly raised” and a statistically significant decrease from pre- to post-test (“close to average”) was found with a medium effect size. Similarly, a statistically significant increase from pre- (“slightly lowered”) to post-intervention (“close to average”) in prosocial scale was revealed with a large effect size. These results indicate that students improved in prosocial behavior (e.g., *considerate of other people’s feelings; often volunteers to help others*) and decreased in peer problems (e.g., *rather solitary, tends to play alone; picked on or bullied*). To our knowledge, only one study measured social variables and also encountered significant improvements in social resiliency (e.g., self-regulation, responsibility, social competence, and empathy) [22]. These results are relevant since social competence has been found to be a key factor in increasing students’ wellbeing [39] and might help prevent the development of more serious social problems such as school bullying [40]. Finally, with respect to life satisfaction, it is first important to note that this measure, which has been related with quality of life and low mental health difficulties [41], was not included in previous studies and, therefore, is novel in the present investigation. In this case, the students’ SWLS scores correspond to a slight dissatisfaction in both pre- and post-intervention. There was a non-significant and small increase in the SWLS from pre- to post-intervention. A possible explanation might be that the students were experiencing the consequences of the COVID-19 pandemic, such as social isolation, reducing positive activities, etc., while receiving the program. Nonetheless, future studies would help to confirm this result.

### Limitations

Despite there being good acceptability and a tendency to improve in several components after the DBT STEPS-A program, it is important to highlight that no statistically significant pre–post-improvements were found in the majority of the effectiveness outcomes and some of them showed small effect sizes. This might be due to a number of limitations. A possible explanation of the results would be that this program was delivered at a universal level. A review on the effectiveness of universal interventions has shown mixed results [11]. In general, health prevention programs applied at a universal level (i.e., for all the students) show more modest treatment-effect sizes than those reported by studies using indicated or selective approaches, as initial levels of symptoms are lower for universal interventions to begin with, so subsequent reductions may be more modest [42], as if there would be more room for improvement if baseline status were more maladaptive [43]. This finding was evident from multiple studies which have reported on universal interventions conducted in schools with children of various ages and with different therapeutic modalities [11] and it is similar to what we found in this sample, that is students with scores that are close to the norm and pre- to post-test small effect sizes for most variables. Working on selecting more reliable measures that capture transdiagnostic dimensions, such us emotional regulation and resilience after universal interventions, might be a future direction to improve this problem. Another factor that might have interfered with the results was the COVID-19 pandemic, which can be seen as an extraordinary and stressful situation with negative consequences (social isolation, deprivation of positive activities, etc.) occurring while students received the program. Replications of this study in a more “normative” environment would be needed to generalize the results. Finally, another shortcoming is that this research consists of a pilot study with a quasi-experimental design. Therefore, it is recommended that future studies further explore these findings with larger sample sizes and adding comparison groups using a randomized controlled trial. 

## 5. Conclusions

Research suggests that the psychological well-being and mental health of adolescents should be the first target of every prevention plan. Emotional dysregulation has been shown to be a key etiology and maintenance factor for mental disorders in youth. DBT skills training has demonstrated good evidence to reduce emotional dysregulation in adolescents. The goal of this study was to evaluate the acceptability and preliminary effectiveness of a DBT skills program for Spanish adolescents in secondary school at a universal level. The general findings show good acceptance of the DBT STEPS-A and that the program could modestly help improve difficulties in emotional regulation, mental health difficulties (specifically social problems), and life satisfaction in Spanish adolescents from the community. Evaluating the effectiveness of the DBT STEPS-A program in adolescents with higher scores on emotional dysregulation at baseline might help confirm if this program has a larger effect specifically on the difficulties in emotional regulation. Future studies including larger samples and control groups would also help replicate these results.

## Figures and Tables

**Table 1:** Adapted DBT STEPS-A curriculum ^1^.

Module	Lessons
Orientation	Lesson 1. Orientation
	Lesson 2. Dialectical principles
Mindfulness	Lesson 3. Goals of mindfulness
	Lesson 4. Mindfulness “what” skills
	Lesson 5. Mindfulness “how” skills
Distress tolerance	Lesson 6. Goals of distress tolerance
	Lesson 7. Crisis survival skills
	Lesson 8. Crisis survival skills
	Lesson 9. Crisis survival skills
	Lesson 10. Reality acceptance skills
	Lesson 11. Reality acceptance skills
	Lesson 12. Mindfulness of thoughts Test 1 (distress tolerance)
Mindfulness	Lesson 13. Mindfulness “what” skills
	Lesson 14. Mindfulness “how” skills
Emotional regulation	Lesson 15. Goals of emotional regulation
	Lesson 16. Naming emotions
	Lesson 17. Managing difficult emotions
	Lesson 18. Managing difficult emotions
	Lesson 19. Reducing vulnerability
	Lesson 20. Reducing vulnerability
	Lesson 21. Mindfulness of emotions
	Lesson 22. Test 2 (emotional regulation)
Mindfulness	Lesson 23. Mindfulness “what” skills
	Lesson 24. Mindfulness “how” skills
Interpersonal effectiveness	Lesson 25. Goals of interpersonal effectiveness
	Lesson 26. DEARMAN
	Lesson 27. GIVE
	Lesson 28. FAST
	Lesson 29. Review of interpersonal effectiveness skills
	Lesson 30. Test 3 (interpersonal effectiveness) and closing

^1^ Adapted from Mazza et al. [19].

**Table 2:** Results of program satisfaction after one academic year of DBT STEPS-A (n = 51).

Questions	Answers	%
1. Do you think the emotional regulation course was useful for your life?	Yes, very	56
	Yes, quite a lot	8
	No, not at all	12
	Prefer not to answer	24
2. Did you enjoy the emotional regulation classes?	Yes, a lot	6
	Yes, quite a lot	56
	No, not much	26
	No, not at all	12
3. Did you use the learned skills since the beginning of the course?	Yes	48
	No	38
	I don’t remember it	14
4. How would you rate the emotional regulation course so far?	(0–4.9) Insufficient	12
	(5–6) Good	40
	(7–8) Remarkable	40
	(9–10) Outstanding	8
5. How would you rate your regulation course teacher?	(0–4.9) Inadequate	0
	(5–6) Good	18
	(7–8) Remarkable	34
	(9–10) Outstanding	48
6. How would you rate yourself based according to your level of… [Class participation]?	(0–4.9) Inadequate	28
	(5–6) Good	36
	(7–8) Remarkable	30
	(9–10) Outstanding	6
7. How would you rate yourself according to your level of… [Attention in class]?	(0–4.9) Inadequate	14
	(5–6) Good	20
	(7–8) Remarkable	48
	(9–10) Outstanding	18
8. How would you rate yourself according to your level of… [Respect for classmates, teachers, and materials]?	(0–4.9) Inadequate	6
	(5–6) Good	8
	(7–8) Remarkable	36
	(9–10) Outstanding	50

**Table 3:** Qualitative questions addressed in the satisfaction survey ^2^.

Questions
What do you like MOST about the emotional regulation classes? What do you like LEAST about the emotional regulation classes? Highlight what aspects of the classes you would improve. This is a space for you to express your opinion. You can explain anecdotes or personal situations that this course has provided to you. You can also explain if you imagined the course would be like this or you can clarify any previous answers.

^2^ Translated from Spanish into English.

**Table 4:** Pre- to post-changes and effect sizes after one academic year of DBT STEPS-A (n = 51).

Variables	Pre-Test M (SD)	Post-Test M (SD)	Z	*p*	Cohen’s d
SWLS_Satisfaction with life	17.07 (4.39)	17.31 (4.25)	−0.147	0.883	−0.05
SDQ_Emotional symptoms	3.85 (2.76)	3.43 (2.59)	−0.968	0.333	0.15
SDQ_Conduct problems	3.04 (1.83)	2.54 (1.88)	−1.548	0.122	0.27
SDQ_Hyperactivity/inattention	5.00 (2.42)	4.87 (2.34)	−0.604	0.546	0.05
SDQ_Peer problems	3.10 (2.20)	2.02 (1.88)	−2.932	0.003 *	0.52
SDQ_Total difficulties	14.08 (6.32)	13.79 (5.81)	−0.356	0.721	0.04
SDQ_Prosocial behavior	6.61 (2.32)	8.22 (1.50)	−3.749	<0.001 *	−0.82
DERS_Emotional inattention	11.54 (4.38)	11.45 (3.68)	−0.038	0.969	0.02
DERS_Emotional rejection	14.15 (7.62)	13.94 (6.83)	−0.018	0.986	0.03
DERS_Emotional uncontrol	19.88 (8.04)	19.25 (9.92)	−0.678	0.498	0.06
DERS_Daily interference	11.15 (4.75)	10.47 (4.74)	−0.622	0.534	0.14
DERS_Total score	65.58 (20.47)	64.43 (22.63)	−0.622	0.534	0.05

Note: SWLS: Satisfaction with life Scale; SDQ: Strengths and Difficulties Questionnaire; DERS: Difficulties in Emotional Regulation Scale; M: mean; SD: Standard Deviation; Z: Wilcoxon test; p: significance level. * *p* ≤ 0.01.

## Data Availability

The data presented in this study are available on request from the corresponding author. The data are not publicly available due to ethical reasons.
